# Crystalline Quality and Surface Morphology Improvement of Face-to-Face Annealed MBE-Grown AlN on h-BN

**DOI:** 10.3390/ma15238602

**Published:** 2022-12-02

**Authors:** Aly Zaiter, Adrien Michon, Maud Nemoz, Aimeric Courville, Philippe Vennéguès, Vishnu Ottapilakkal, Phuong Vuong, Suresh Sundaram, Abdallah Ougazzaden, Julien Brault

**Affiliations:** 1 Université Côte d’Azur, CNRS-CRHEA, French National Center for Scientific Research, 06560 Valbonne, France; adrien.michon@crhea.cnrs.fr (A.M.); maud.nemoz@crhea.cnrs.fr (M.N.); aimeric.courville@crhea.cnrs.fr (A.C.); philippe.vennegues@crhea.cnrs.fr (P.V.); 2 French National Center for Scientific Research, IRL 2958 Georgia Tech, 2 rue Marconi, 57070 Metz, France; vishnu.ottapilakkal@georgiatech-metz.fr (V.O.); pvuong@georgiatech-metz.fr (P.V.); suresh.sundaram@georgiatech-metz.fr (S.S.); abdallah.ougazzaden@georgiatech-metz.fr (A.O.); 3 Georgia Institute of Technology, School of Electrical and Computer Engineering, Atlanta, GA 30332-0250, USA; 4 Georgia Tech-Lorraine, 2 rue Marconi, 57070 Metz, France

**Keywords:** molecular beam epitaxy, nitrides, wide bandgap materials, aluminum nitride, boron nitride, postgrowth annealing, X-ray diffraction, atomic force microscopy, transmission electron microscopy

## Abstract

In this study, AlN epilayers were grown by ammonia-assisted molecular beam epitaxy on 3 nm h-BN grown on c-sapphire substrates. Their structural properties were investigated by comparing as-grown and postgrowth annealed layers. The role of annealing on the crystalline quality and surface morphology was studied as a function of AlN thickness and the annealing duration and temperature. Optimum annealing conditions were identified. The results of X-ray diffraction showed that optimization of the annealing recipe led to a significant reduction in the symmetric (0 0 0 2) and skew symmetric (1 0 −1 1) reflections, which was associated with a reduction in edge and mixed threading dislocation densities (TDDs). Furthermore, the impact on the crystalline structure of AlN and its surface was studied, and the results showed a transition from a surface with high roughness to a smoother surface morphology with a significant reduction in roughness. In addition, the annealing duration was increased at 1650 °C to further understand the impact on both AlN and h-BN, and the results showed a diffusion interplay between AlN and h-BN. Finally, an AlN layer was regrown on the top of an annealed template, which led to large terraces with atomic steps and low roughness.

## 1. Introduction

Solid-state ultraviolet (UV) light-emitting diodes (LEDs) exhibit better features (e.g., compactness, broad wavelength emission range, and low power consumption) compared to mercury lamps, which are toxic, high-voltage, and bulky [1]. Due to its wide direct bandgap of 6.2 eV, high chemical and thermal stability, high thermal conductivity, high surface acoustic wave velocity, low thermal expansion coefficient, and high breakdown dielectric strength [1,2,3], aluminum nitride (AlN)-based materials have drawn significant interest for use in aluminum gallium nitride (AlGaN)-based UV LEDs, which have a number of therapeutic and diagnostic medical applications, e.g., UV curing, disinfection of medical tools, purification of microbiological contaminants in water and air, and biomedical and analytical instrumentation [1,4,5].

Several growth techniques are used to grow high-quality AlN layers, such as metalorganic vapor phase epitaxy (MOVPE), pulsed-laser deposition (PLD), and molecular beam epitaxy (MBE). MBE growth technique has several advantages. It uses low growth temperatures that limit interdiffusion and phase segregation, has the ability to accurately control the deposited thickness at the atomic scale, uses ultrahigh-purity source materials, and enables in situ observation of the surface structure by reflection high-energy electron diffraction (RHEED) [3]. Regarding the importance of the substrate in epitaxial growth, native AlN bulk substrates present unique features, such as high crystal quality and remarkably low threading dislocation densities (TDDs) (<10^5^ cm^−2^). However, they are expensive, scarce, and come in small sizes (typically below 2 inches). Sapphire is mostly used as a substrate of choice for the growth of AlN due to its low cost, transparency in the UV range, and large size availability (up to 8 inches). However, despite these advantages, some drawbacks are also present, such as a high lattice mismatch (~13% for AlN for the basal plane lattice parameter, a_AlN_ = 3.112 Å) and a lattice thermal coefficient mismatch (~43% for the basal plane), which induce several epitaxial challenges. Indeed, these mismatches decrease the crystalline structural quality of the epitaxial layer and increase the TDDs, which act as nonradiative recombination centers and thereby limit the efficiency of LEDs.

Postgrowth annealing is one of the solutions to improve the crystalline structure quality of AINs [6,7]. In particular, Miyake et al. developed the use of face-to-face high-temperature annealing (FFA) in nitrogen ambience on sputtered and MOVPE-grown AlN on sapphire [8]. They were able to reduce the TDDs and modify the surface morphology from a columnar aspect to a step and terrace one, all the while improving the crystalline structural quality [8]. Another solution that has attracted wide interest to circumvent the lattice mismatch issue is the use of two-dimensional (2D) materials [9]. Their extended crystalline planar structures, which are held together by strong in-plane covalent bonds and weak out-of-plane van der Waals forces, make it possible for their individual layers to be easily removed by breaking the van der Waals bonds with little damage either to the extracted layer or to the remaining structure [10]. Therefore, van der Waals epitaxy enables these special features of 2D materials to be exploited to significantly reduce the lattice matching problem faced in the conventional growing methods of heterostructures [9]. Although many 2D materials could be potentially used, hexagonal boron nitride (h-BN) seems to be among the best suited due to its chemical compatibility with AlN- or AlGaN-based epitaxial layers. In addition, h-BN templates can serve as mechanical release layers for III-nitride devices, such as LEDs [11,12], to be transferred to adequate substrates, therefore creating flexible devices [11]. However, the growth of high-quality III-nitride films is not straightforward as the lack of dangling bonds at the surface complicates the nucleation step as in the case of gallium nitride (GaN) growth on h-BN, which can result in the formation of randomly oriented, polycrystalline, and isolated islands [11]. Meanwhile, due to the lower mobility and higher sticking coefficient of Al adatoms, AlN could serve as a better nucleation layer on h-BN. 

Limited studies exist regarding the improvement of AlN quality on h-BN, and most of these studies used MOVPE. Dipankar Chugh et al. improved the morphology and crystal quality of AlN grown on h-BN by MOVPE by adopting a modified multilayer process involving pulsed ammonia flow for depositing smooth AlN layers on h-BN/sapphire templates [13]. Qingqing Wu et al. reported the growth of monolayer h-BN by low-pressure chemical vapor deposition (CVD) on copper (Cu) foil substrate and then transferred it on a sapphire substrate for the growth of AlN and DUV LEDs [14]. The same group also reported crack-free crystalline AlN and DUV LEDs with an emission at 281 nm on MOVPE-grown multilayer h-BN, thus showing the advantage of multilayer MOVPE-grown h-BN to obtain good-quality epilayers and devices on large surfaces [12]. On the other hand, the growth of AlN on h-BN by MBE has not yet been reported and will be investigated in this study.

In this work, the fabrication of AlN layers by MBE on h-BN/sapphire was carried out. In addition, we studied the effects of postgrowth FFA on the surface morphology and crystalline quality of the AlN epitaxial layers. The h-BN, with a thickness of 3 nm, was directly grown on 2-inch sapphire substrates by MOVPE.

The annealing role on h-BN and AlN structural properties was investigated as a function of AlN thickness and the annealing duration time. The impact of annealing on the structural properties of both AlN and h-BN materials was investigated. It was found that by adopting a specific annealing recipe, a significant improvement in the crystalline quality of AlN was achieved. In addition, diffusion interplay between AlN and h-BN was also observed. Finally, an AlN layer was regrown by MBE on the top of an annealed AlN/h-BN template showing large terraces with atomic step surface morphology separated by 1–4 nm high steps, affirming the potential use of such templates for the fabrication of AlGaN-based heterostructures.

## 2. Materials and Methods

The growth of 3 nm thick h-BN was performed in an Aixtron MOVPE close-coupled showerhead (CCS) reactor (AIXTRON Company, Cambridge, UK) on (0001) sapphire substrates at 1280 °C and 90 mbar pressure. Triethylboron (TEB) and ammonia (NH_3_) were used as B and N precursors, respectively. Detailed growth conditions of h-BN are reported elsewhere [15]. The AlN template layers were then grown on h-BN by ammonia-assisted MBE using conventional Al effusion cell, NH_3_ as a nitrogen precursor, and a graphite furnace designed for high temperatures in a RIBER 32P reactor (RIBER S.A, Bezons, France). The procedure used for the growth of AlN samples is as follows. An AlN buffer layer of 5 nm was first grown at 950 °C with an ammonia flow rate of 50 sccm. Next, AlN growth was performed at 1050 °C with an ammonia flow rate of 50 sccm and at a growth rate of 100 nm/h. We considered two templates with different AlN layer thicknesses: sample A (50 nm) and sample B (100 nm).

For the annealing process, a home-made resistively heated horizontal hot wall CVD reactor with a graphite chamber was used. The surface of sample B was covered with the surface of sample A in a face-to-face configuration to suppress the thermal decomposition of the AlN films during annealing. The annealing steps were conducted at temperatures ranging from 1450 to 1650 °C under a constant N_2_ laminar flow of 6 slm (standard liters per minute) and a total pressure of 800 mbars. Right beneath the samples, a pyrometer focused on the substrate holder enabled us to monitor the temperature. After the temperature rise and its stabilization, the samples were left under N_2_ carrier gas for a duration of 15 min for each annealing. Then, when the heating was terminated, the samples were left to cool down to room temperature under the carrier gas (at an initial cooling rate of 3 °C/s).

Atomic force microscopy (AFM) EDGE-DIMENSION (BRUKER, Billerica, MA USA), operating in tapping mode was used to investigate the surface morphology, and data were processed using WSxM software (version number 4.0 Beta 9.1) [16]. In addition, in order to study the crystalline quality of the AlN layers and to investigate the influence of annealing, X-ray diffraction (XRD) measurements were performed using a PANalytical X’Pert PRO MRD four-circle diffractometer (Malvern Panalytical, Malvern, United Kingdom). Omega scans of both the symmetric (0 0 0 2) and skew symmetric (1 0 −1 1) plane reflections were measured. Cross-sectional transmission electron microscopy (TEM) (Thermos Fisher Company, Massachusetts, United States) was also performed on sample A after all the annealing steps. Specimens for transmission electron microscopy (TEM) were prepared using a conventional technique involving mechanical thinning followed by ion milling using Ar^+^ at 0.5–4.5 keV. The samples were observed using a Titan 80-300 microscope (CP2M, Marseille, France).

Finally, in order to assess whether annealing could be used to fabricate layers of AlN as ready-to-grow templates for the fabrication of AlN-based structures, a regrowth step by MBE of 220 nm AlN at 1100 °C (sample C) on sample B (annealed 100 nm thick AlN template) was carried out. To assess this growth process, AFM and XRD measurements were followed to study the surface morphology and the crystalline structure.

## 3. Results

### 3.1. Growth of AlN by MBE

#### 3.1.1. Characterization of h-BN Templates before Growth

**Figure 1:**
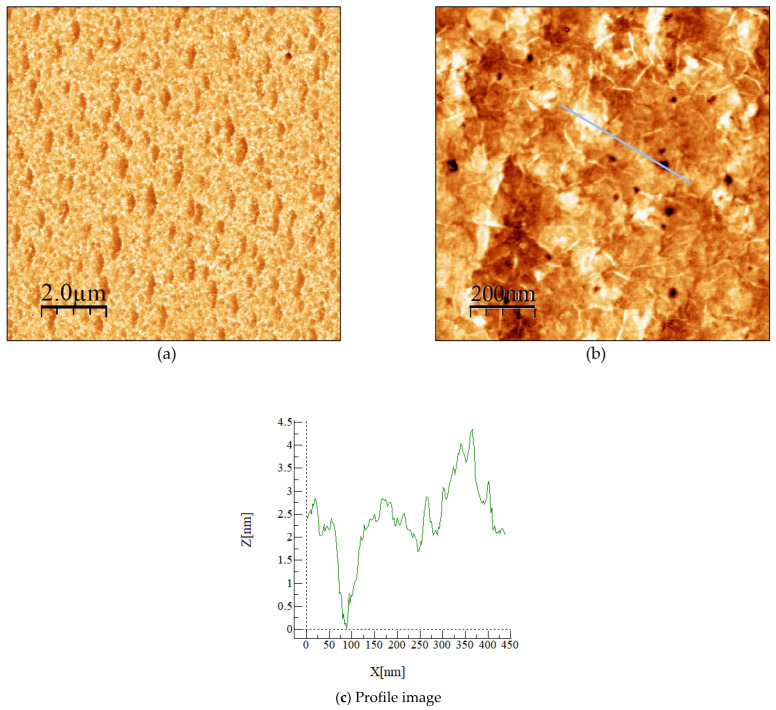
Atomic force microscopy images of 3 nm h-BN epilayer grown on sapphire c-plane substrate. (**a**) (10 × 10) μm^2^ image with an RMS = 0.7 nm, (**b**) (1 × 1) μm^2^ image with an RMS = 0.5 nm, and (**c**) profile measurement of a specific marked zone indicated by a blue line in (**b**).

As a first step, the two 3 nm thick h-BN layers on sapphire samples were characterized to investigate their surface morphology. AFM topographic images of (10 × 10) µm^2^ and (1 × 1) µm^2^ together with their root mean square (RMS) values are reported in Figure 1a,b.

Figure 1a shows the h-BN layer that covers the entire wafer surface with a measured surface RMS roughness of 0.7 nm. Figure 1b presents a 1 × 1 µm^2^ AFM scan that shows small holes (with a density of 1.9 × 10^8^ cm^−2^) covering the entire surface. These holes can be described as pits of hexagonal shape with a depth of 2.5 ± 0.5 nm (Figure 1c). The surface RMS roughness was measured as 0.5 nm. The presence of wrinkles (white segments in the AFM image) is typical for 2D materials in general. In the case of h-BN, it is due to the thermal expansion coefficient (TEC) difference between h-BN and sapphire substrates, which induces a compressive strain in the layer during the cooling process. The wrinkling instability releases the energy, which creates the roughness in the sample [15,17,18]. The observation of wrinkles is an indicator of the high structural quality of the layered h-BN.

#### 3.1.2. Growth of AlN on h-BN/Sapphire Templates by MBE

**Figure 2:**
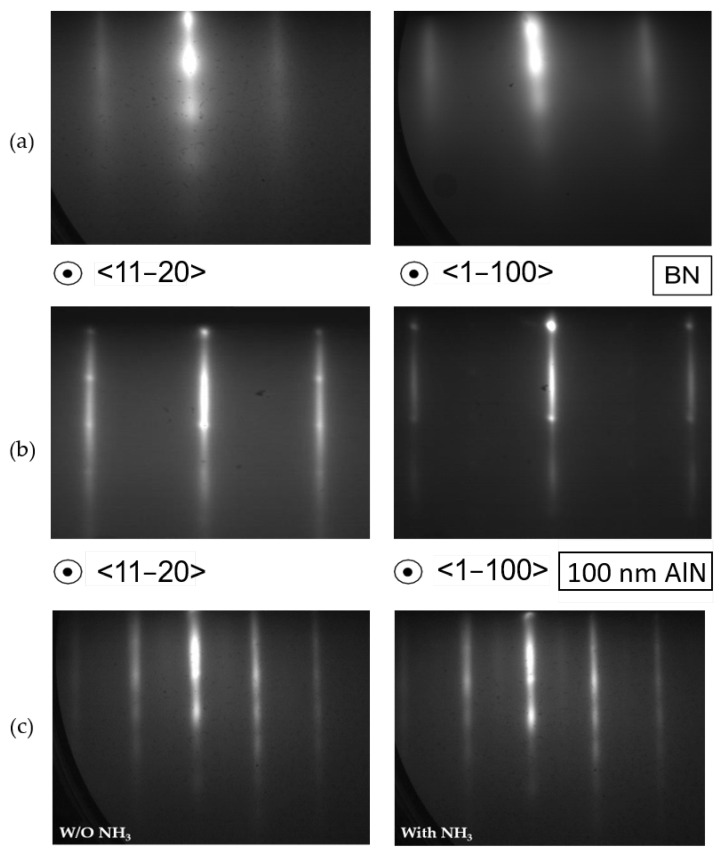
Reflection high-energy electron diffraction (RHEED) images along the 〈1 1 −2 0〉 and 〈1 −1 0 0〉 azimuths of the surface. (**a**) The h-BN layer, (**b**) the AlN surface at the end of the growth. The spots were attributed to the presence of islands (i.e., 3D-like structures) on the surface, while the dim and broadened patterns were caused by charge effects due to the insulating nature of the h-BN and AlN surface. (**c**) Images of the diffraction of the AlN surface after the cooling down process without NH_3_ (left) and under NH_3_ (right) (for a substrate temperature <550 °C). The 2 × 2 reconstruction confirmed the Al polarity of the layer.

We proceeded to grow AlN on both h-BN samples by MBE. Figure 2a shows RHEED images of the h-BN surface before growth along the <1 1 −2 0> (left) and <1 −1 0 0> (right) azimuths. Charge effects due to the insulating nature of the h-BN surface caused the dim and broadened patterns observed. Figure 2b shows the diffraction pattern on sample B (100 nm thick AlN) after growth. The smooth vertical lines observed were an indication of the smoothness of the surface morphology. Moreover, the spots were attributed to the presence of islands (i.e., 3D-like structures) on the surface. Studying the surface reconstruction by RHEED can be a way to determine the polarity of the AlN layers. After finishing the growth of AlN and decreasing the surface temperature under NH_3_ flux, it was possible to observe a transition from 1 × 1 to 2 × 2 surface reconstruction when the temperature went below 600 °C. For NH_3_-assisted MBE, it is a characteristic feature of the metal polarity of the nitride layer [19,20]. Figure 2c displays the diffraction patterns of the AlN surface after growth at low temperature (<550 °C) and the 2 × 2 reconstruction under NH_3_, which confirmed the Al polarity of the layer.

### 3.2. Postgrowth High-Temperature Annealing

**Figure 3:**
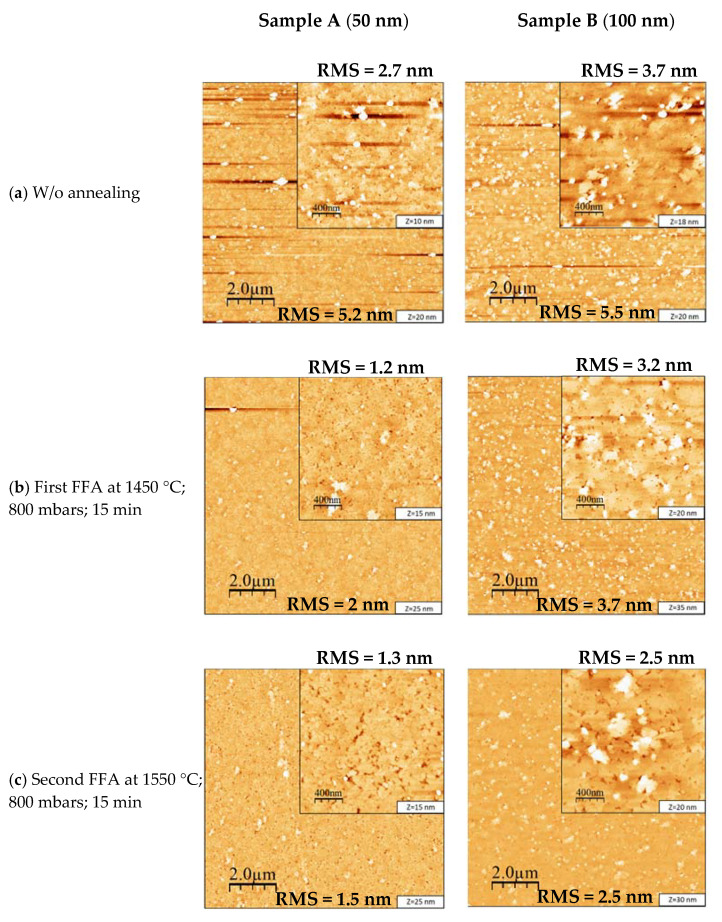

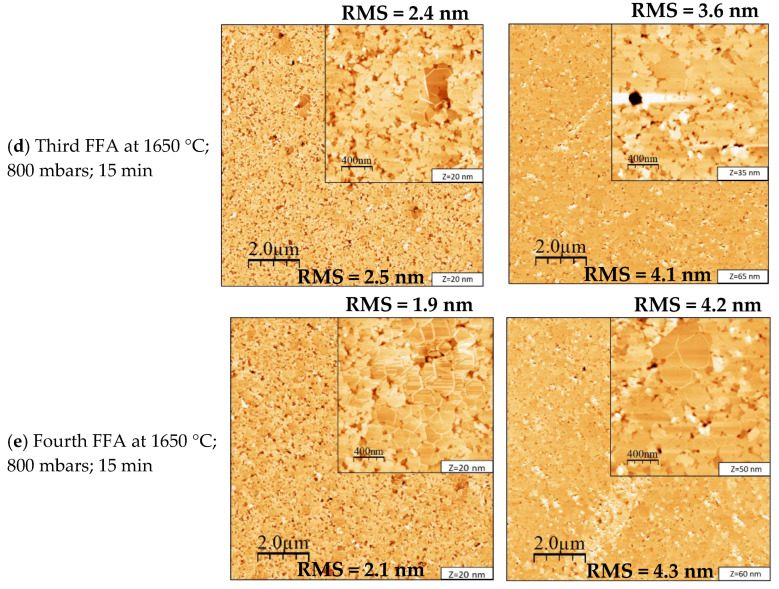
Atomic force microscopy images of (10 × 10) µm^2^ of samples A and B, i.e., 50 and 100 nm of AlN grown on 3 nm h-BN, respectively: (**a**) without postgrowth annealing, (**b**) with first annealing at 1450 °C, (**c**) with second annealing at 1550 °C, (**d**) with third annealing at 1650 °C, and (**e**) with fourth annealing at 1650 °C. The insets show 2 × 2 μm^2^ scan images. The term Z represents the vertical scale and the variation in height in the AFM images, and its value is reported at the bottom right hand side of each image.

Figure 3 shows the AFM images ((10 × 10) and (2 × 2) µm^2^) for samples A (left) and B (right) before and after FFA. In Figure 3a for sample A, a rough surface (RMS = 5.2 nm) defined by islands (density around 3.3 × 10^8^ cm^−2^, height ~50 ± 15 nm, width ~170 ± 27 nm) was observed. Figure 3b–e shows AFM images after FFA (images on the left side for sample A). First, the image after annealing at 1450 °C is shown in Figure 3b, followed by the second annealing at 1550 °C in Figure 3c, and then the third and fourth annealing steps at 1650 °C in Figure 3d,e, respectively. As can be seen, after the first FFA, the surface got smoother with a decrease in the island height (~25 ± 4 nm) and width (~100 ± 12 nm) with an RMS roughness of 2 nm. However, the density of the islands remained constant (at around 3 × 10^8^ cm^−2^). For the second annealing at 1550 °C. As can be observe from Figure 1c, the islands decreased significantly down to a density of 1.2 × 10^8^ cm^−2^, and the RMS roughness decreased to 1.5 nm. In addition, holes could be clearly observed and appeared to be larger as the annealing temperature increased. They were actually already present, but their characteristics were difficult to be determined due to the surface roughness. Figure 3d shows AFM images after the third annealing at 1650 °C, where the surface roughness RMS had increased from 1.5 to 2.5 nm. As shown in Figure 3e, after the fourth annealing at 1650 °C, an additional material was clearly observed on the surface. In fact, its presence could already be observed after the third annealing, as shown in the inset of Figure 3d. Compared to the previous annealing, the surface roughness RMS had decreased to 2.1 nm.

As shown in Figure 3a, for sample B before FFA, a rough surface defined by a high density of 3D islands of about 6.9 × 10^8^ cm^−2^ could be seen. The RMS roughness was 5.5 nm; the average island height was 53 ± 11 nm, which was somewhat similar to the islands height of sample A; and the average width was 120 ± 19 nm. Figure 3b–e shows AFM images after FFA. After the first FFA, as can be observed, the surface got smoother with a decrease in the island density down to 5.5 × 10^8^ cm^−2^. The size also decreased, with the height being around 37 ± 4 nm and the width around 100 ± 20 nm. Consequently, the RMS roughness was found to decrease with a value of 3.7 nm. For the second annealing at 1550 °C, as can be observed from Figure 3c, the island density and size continued to decrease, with the density going down to 3.8 × 10^8^ cm^−2^ and the height decreasing to ~15 ± 4 nm. Accordingly, the RMS also decreased to 2.5 nm. At this stage, holes started to have a clearer appearance on the surface after the reduction of the presence of islands, similar to sample A. Figure 3d shows the third annealing at 1650 °C, where the 3D islands have mostly disappeared. However, the surface got rougher, with the RMS roughness increasing from 2.5 to 4.1 nm. As can be seen in Figure 3e, the fourth annealing at 1650 °C increased the roughness of the surface up to an RMS value of 4.3 nm. Similar to the case of sample A, an additional material was observed in small regions of the sample’s surface. Notably, its presence was more significant on sample A, as can be observed in the inset of Figure 3d after the third annealing. This difference between samples A and B was attributed to the difference in thickness of the AlN layer.

**Figure 4:**
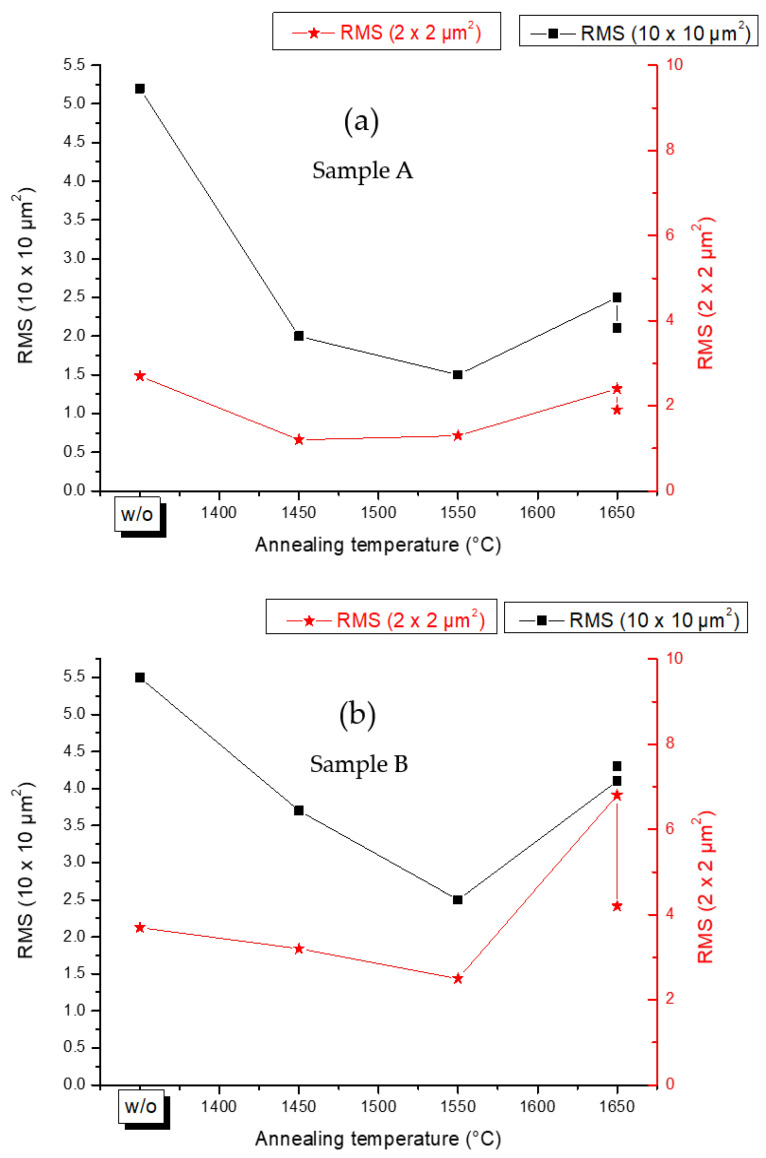
Variation of the RMS roughness as a function of the annealing temperatures for (**a**) sample A and (**b**) sample B. The RMS values are measured on (10 × 10) μm^2^ atomic force microscopy topographic images.

Figure 4 illustrates the RMS roughness variation of samples A and B before and after each FFA stage. As can be observed, the FFA positively affected the RMS roughness of the AlN surface of both samples by initially decreasing and eliminating the 3D island structures. However, after the third annealing at 1650 °C, an increase in the RMS roughness was evidenced, which was related to the presence of holes and the formation of an additional material at the surface of the AlN layer.

The omega scan FWHM are impacted by many factors, such as the wafer curvature, instrument width, coherence length, lattice strain, and lattice rotation due to dislocations (tilt and twist misorientations). For films with high TDDs, as in our case here, the lattice rotations due to dislocations have the most important influence [20,21,22]. Therefore, the tilt, defined as out-of-plane misorientation, and the twist, defined as in-plane misorientation, can be evaluated from the symmetric and skew symmetric reflections. XRD rocking curve full width at half maximum (FWHM) of symmetric (0 0 0 2) and skew symmetric (1 0 −1 1) diffractions for both samples were studied (the data is provided as supplementary material (check Figures S1–S3 in Supplementary Material)). By coupling the XRD measurements with the Dunn and Koch equation [23], it was then possible to correlate mixed and edge TDDs with the FWHM of the symmetric and skew symmetric reflections [6,23]. It is critical to mention that the screw TDDs were neglected as they are present in very low proportion (~1%) in nitrides [24].

**Figure 5:**
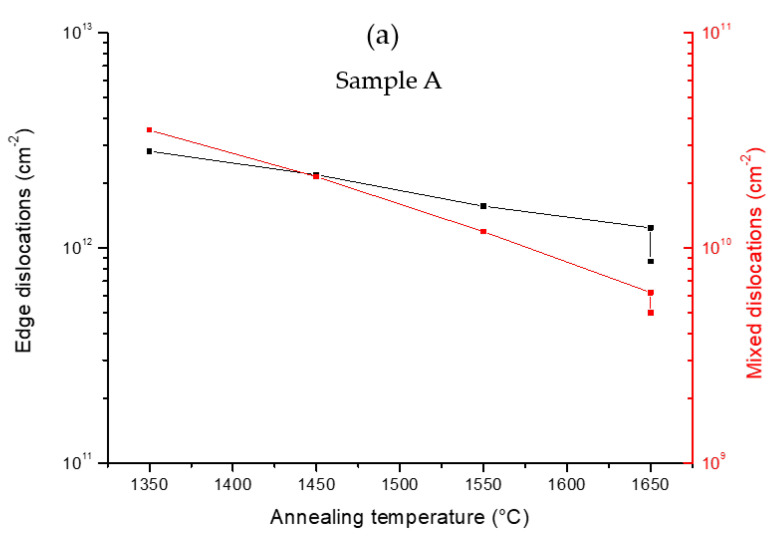

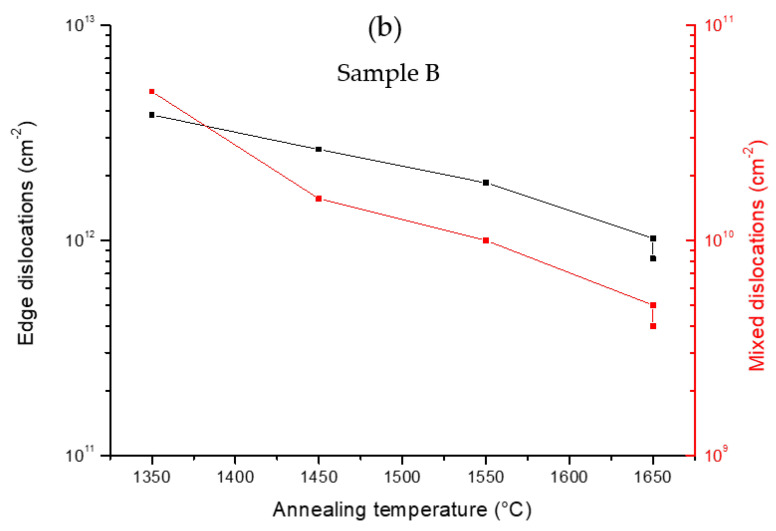
Mixed and edge dislocation densities of (**a**) sample A and (**b**) sample B estimated from tilt and twist values as a function of the annealing temperatures.

The estimation of mixed and edge TDDs for samples A and B are shown in Figure 5a,b, respectively. For sample A, the mixed TD density before the FFA was in the 4 × 10^10^ cm^−2^ range, and it decreased by a factor of 8 to 5 × 10^9^ cm^−2^ after the complete FFA process. Regarding the edge TD density, a very high density of 3 × 10^12^ cm^−2^ was estimated before the FFA, which then decreased down by a factor of 3 to 9 × 10^11^ cm^−2^ after FFA. For sample B, the mixed TD density before the FFA was 5 × 10^10^ cm^−2^, and it decreased to 4 × 10^9^ cm^−2^ after the FFA, corresponding to an improvement by a factor of 10. At the same time, the edge TD density went from 4 × 10^12^ cm^−2^ before the FFA down by a factor of 5 to 8 × 10^11^ cm^−2^ after annealing. These results indicated an improvement of the structural quality of the AlN film due to reduction in the tilt and twist components from the epitaxial AlN films by high-temperature annealing.

### 3.3. AlN Regrowth by MBE

**Figure 6:**
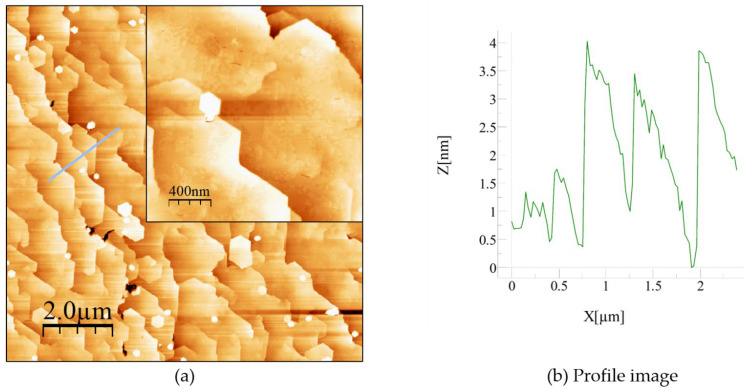
Atomic force microscopy images of sample C, a 220 nm thick AlN epilayer grown on annealed sample B. (**a**) (10 × 10) μm^2^ image with an RMS = 1.7 nm, the inset showing (2 × 2) μm^2^ scan image with an RMS = 1 nm. (**b**) Profile measurement of a specific marked zone of the (10 × 10) µm^2^ image indicated by a blue line.

We then proceeded to regrow a 220 nm thick AlN layer (sample C) on sample B (annealed 100 nm thick AlN) to investigate the influence of the annealed template on the crystalline quality and the surface morphology of the regrown AlN layer. Once again, the growth was performed using MBE with ammonia (NH_3_) as the nitrogen source. Figure 6 shows the AFM images of sample C, which indicate a smoother surface morphology compared to samples A and B. The surface was made of large terraces with the presence of atomic steps and nm-high steps separating each terrace. For the (10 × 10) µm^2^ surface image, an RMS roughness of 1.7 nm was formed. The morphology was made of terraces with an average terrace length of 920 ± 110 nm and separated by steps with an average height of 3.5 ± 0.5 nm, i.e., corresponding roughly to 14 monolayers of AlN.

In addition, for the (2 × 2) µm^2^ surface image, the RMS roughness was reduced to 1 nm. The AFM results clearly showed how the annealing recipe ameliorated the surface morphology of the AlN layer by developing large terraces with smooth atomic step morphology. In addition, for both the symmetric plane (0 0 0 2) and the skew symmetric plane (1 0 −1 1), the crystalline structure study by X-ray measurements showed a slight reduction in the FWHM values; however, this was found to fall within the instrumental measurement precision. The edge and mixed TDDs values were estimated to show a total reduction from 4 × 10^12^ cm^−2^ to 7 × 10^11^ cm^−2^ and from 5 × 10^10^ cm^−2^ to 4 × 10^9^ cm^−2^ between the initial AlN layer and the final one obtained after the HTA process and AlN regrowth, respectively. This clearly shows that such annealed MBE-grown AlN templates can be used as “ready-to-grow” templates for the fabrication of AlN-based structures with better surface morphology.

The estimated edge and mixed TDDs are summarized in Table 1 along with the values of the RMS roughness and the FWHM of the symmetric and skew symmetric planes. We observed that the FFA played a significant role in reducing both the edge and mixed TD densities for samples A and B, thus improving the crystalline qualities of the layers. Sample A’s mixed TD density decreased by 85% in total, while the edge TD density reduced by a total of 70%. Sample B’s mixed TD density had a total reduction of 92%, while its edge TD density decreased by 80%.

## 4. Discussion

**Figure 7:**
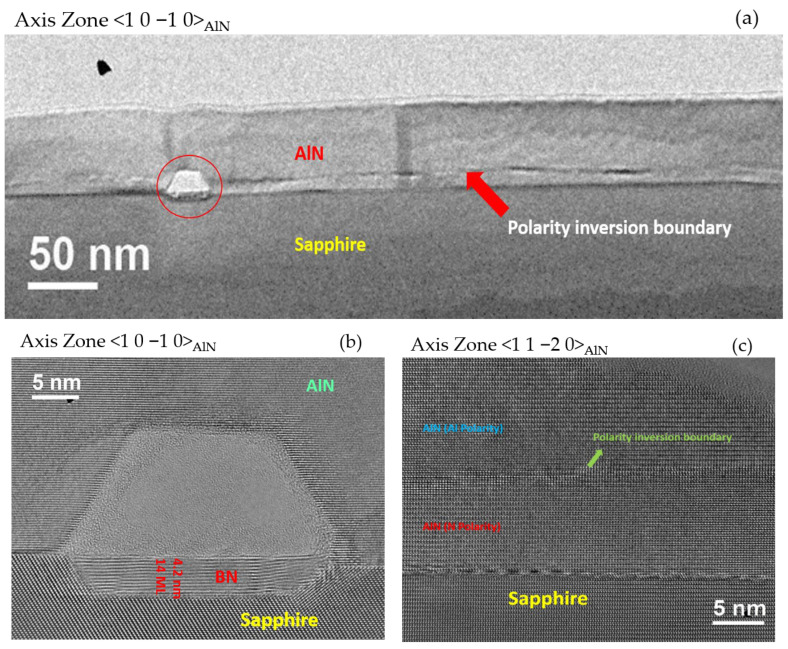
Multibeam cross-sectional TEM images of sample A (50 nm AlN) along the <1 0 −1 0> and <1 1 −2 0> AlN zone axes. (**a**) Low-resolution image of AlN on h-BN showing a cavity at the interface with the sapphire substrate. In addition, a polarity inversion boundary is shown close to the interface (~10 nm above it). (**b**) High-resolution image of the cavity observed in (**a**), where h-BN inclusion in the sapphire zone can be observed. (**c**) High-resolution image of the interface between sapphire and AlN, where h-BN has completely disappeared from the interface with sapphire. In addition, the polarity inversion boundary can be observed with N-polar AlN underneath it and Al-polar AlN above it.

The growth of AlN on h-BN/sapphire templates by MBE led to the fabrication of Al-polar layers with mainly 2D surfaces perturbed by the presence of 3D islands. In order to investigate the surface morphology and crystalline quality, which was characterized by a high degree of mosaicity, a TEM investigation of sample A (50 nm AlN) was performed in the cross section after the whole series of annealing processes were carried out. Figure 7 presents the TEM images along the [1 0 −1 0] and [1 1 −2 0] AlN zone axes. At first, an interface was observed close to the interface between the sapphire and AlN/h-BN (around 10 nm from the interface), which should correspond to an inversion domain boundary between a bottom N-polar and a top Al-polar region, as always reported for face-to-face annealing of AlN on sapphire [25,26]. Moreover, a truncated pyramid-like cavity was found. Figure 7b shows the truncated pyramid-like cavity, shown in Figure 7a, at high resolution. This cavity revealed an unexpected process, with a BN inclusion being found in the sapphire substrate (with an in-depth thickness penetration of around 4.2 nm). The formation of this inclusion was due to the high-temperature annealing. Another consequence of the annealing processes is shown in Figure 7c. As can be seen, no h-BN was found in between the sapphire and AlN layer, i.e., B had diffused into the AlN. In fact, similar observations were found in most of the interface between the nitride layers and the sapphire substrate, and h-BN was found only around cavities, as shown in Figure 7b. In addition, the polarity inversion boundary, indicated in Figure 7a, could be clearly seen with the N-polar AlN underneath it (i.e., at the interface with the sapphire substrate) and the Al-polar AlN above it towards the surface.

**Figure 8:**
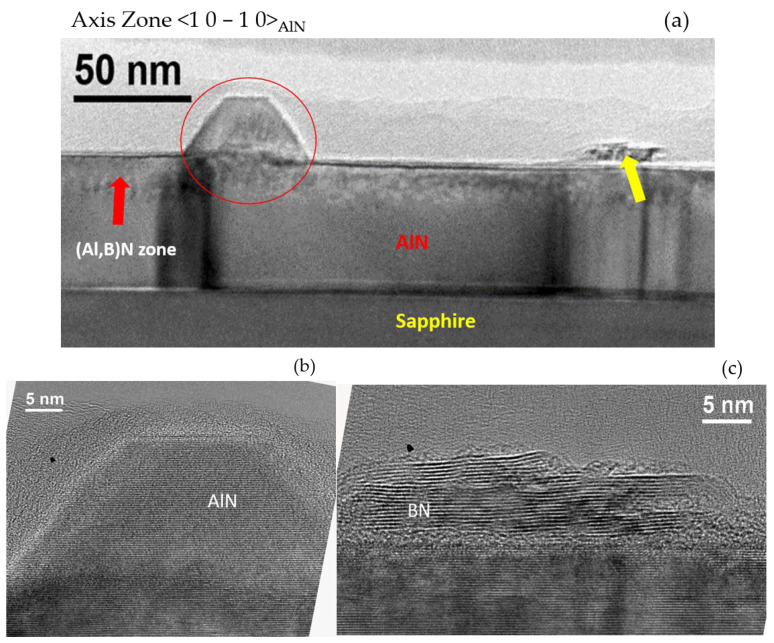
(**a**) AlN truncated pyramid-like structure at the surface (red circle). The yellow arrow points to a region made of h-BN due to the diffusion of B into the AlN layer, which reached the surface and recrystallized after annealing. The red arrow shows a specific contrasted region underneath the surface, which can be attributed to the presence of B in the AlN layer forming an (Al, B)N material. (**b**,**c**) High-resolution images of AlN truncated pyramid-like structure and recrystallized h-BN region, respectively.

**Figure 9:**
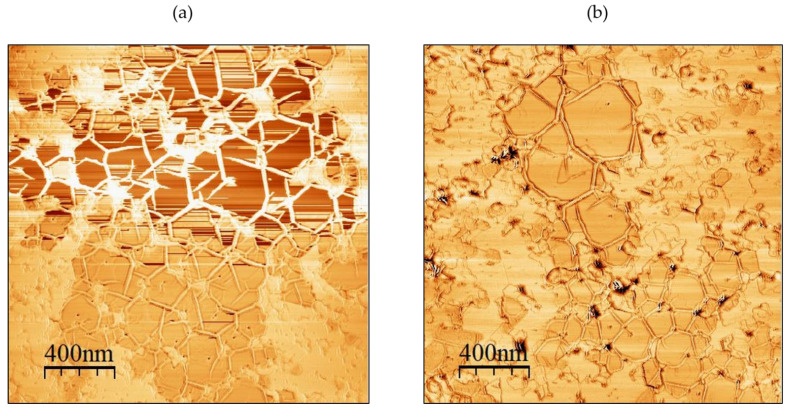
Atomic force microscopy phase images of (2 × 2) µm^2^ showing h-BN regions resulting from the diffusion of h-BN through the AlN up to the surface for (**a**) sample A (50 nm AlN) and (**b**) sample B (100 nm AlN).

Figure 8a shows an AlN truncated pyramid-like structure at the surface, which confirms that the 3D islands observed by AFM before and during annealing were AlN islands. Furthermore, the additional material observed by AFM on the surface of both samples after annealing was found to be BN as its morphology was the characteristic morphology of h-BN. This demonstrated that B had diffused from the interface with sapphire to the surface of the layer and had recrystallized to form h-BN islands (yellow arrow). This was also confirmed by performing AFM phase imaging on both samples (Figure 9). The zone marked by a red arrow is therefore supposed to be a region made of (Al, B)N, which was formed due to the diffusion of B into the AlN layer during annealing. Figure 8b,c are high-resolution images of specific features present at the surface, namely, the AlN truncated pyramid-like structure and the recrystallized h-BN, respectively.

During high-temperature annealing, solid-phase reactions occurred that improved the crystalline quality of the structure, similar to what has been previously reported [8]. It should be mentioned that for both samples (A and B) for the third and fourth annealing steps at 1650 °C, the FWHMs for both the symmetric (0 0 0 2) and skew symmetric (1 0 −1 1) planes hardly decreased (especially for sample B). This can be explained by the study conducted by Prof. Miyake’s group on FFA of AlN films, which showed that the XRD peak FWHM values were similar for annealing carried out at 1600 and 1650 °C and only significantly decreased for annealing at the higher temperature of 1700 °C [8]. This was due to the columnar structure of AlN. The alignment of the AlN columns composing the AlN layer requires high energy to enable coalescence, which then results in annihilation of the domain boundaries and thus improves the crystalline quality of the AlN films [8]. However, in our case, the surface became thermally unstable above 1550 °C annealing temperature. This feature can be explained by the diffusion of B in the AlN layer, which led to the formation of h-BN regions at the surface of the AlN layer. This also clarifies the origin of the increase in RMS after the third annealing and sets a limit for the maximum annealing temperature that can be used.

## 5. Conclusions

AlN epitaxial layers (50 nm and 100 nm thick) were grown on 3 nm thick h-BN/sapphire templates by MBE, and their main structural properties were characterized by AFM and XRD. Next, the influence of postgrowth FFA high-temperature annealing on these AlN structures was studied as a function of the annealing conditions. The temperature was varied from 1450 to 1650 °C, and different annealing time durations were used. The surface roughness (RMS) was determined by AFM, and a significant decrease in AlN thickness was found after the first two annealing steps at 1450 and 1550 °C. Some modifications were observed at the AlN surface, including a decrease in the 3D island structures that were initially present after MBE growth as well as surface smoothening, with the RMS roughness varying from 5.2 to 2.1 nm. However, an observable increase in the RMS roughness after the third and fourth annealing steps at 1650 °C was found. For the (0 0 0 2) and the (1 0 −1 1) reflections, the omega scan FWHM significantly decreased as the annealing temperature increased, indicating the crystalline quality improvement of the AlN layers. Edge and mixed TDDs were estimated and showed a reduction for each annealing step. TEM observations performed after the whole annealing cycle showed that B had diffused from the sapphire interface through the AlN layer. In some regions, the h-BN was even diffused up to the surface, where it had recrystallized on top of the AlN layer. In other zones along the interface with sapphire, h-BN was also found to exist inside the sapphire substrate. These unexpected diffusion mechanisms indicate that annealing conditions should be carefully controlled in order to not reach the thermal instability of h-BN. Finally, an AlN layer was regrown by MBE on the thermally annealed 100 nm thick layer, showing a step bunching morphology with µm-wide terraces. In addition, better structural quality with a reduction in mixed and edge TDDs compared to the initial AlN layers was observed. This study indicates that annealed MBE-grown AlN layers on h-BN could potentially be used as templates for AlN-based heterostructures with improved structural quality by adequately adjusting the annealing temperature and time duration. Furthermore, high-temperature annealing could also open the possibility of (Al,B)N alloy fabrication by B diffusion in AlN.

## Figures and Tables

**Table 1:** Table summarizing the main crystalline and morphological properties of samples A, B, and C measured by atomic force microscopy and X-ray diffraction before and after FFA and after an AlN regrowth step.

	Annealing Temperature (°C)	RMS (nm) (10 × 10) µm^2^	(0 0 0 2) (°)	(1 0 −1 1) (°)	Estimated Mixed TDD (cm^−2^)	Estimated Edge TDD (cm^−2^)
Sample A (AlN 50 nm)	W/O	5.2	0.93	5.67	4.0 × 10^10^	3.0 × 10^12^
	1450	2.0	0.73	5.50	2.1 × 10^10^	2.2 × 10^12^
	1550 1650 1650	1.5 2.5 2.1	0.54 0.39 0.35	4.60 3.77 3.14	1.2 × 10^10^ 6.2 × 10^9^ 5.0 × 10^9^	1.6 × 10^12^ 1.2 × 10^12^ 9.0 × 10^11^
Sample B (AlN 100 nm)	W/O 1450 1550 1650 1650	5.5 3.7 2.5 4.1 4.3	1.10 0.62 0.48 0.35 0.31	6.62 5.01 4.22 3.42 3.06	5.0 × 10^10^ 1.6 × 10^10^ 1.1 × 10^10^ 5.1 × 10^9^ 4.0 × 10^9^	4.0 × 10^12^ 2.6 × 10^12^ 1.8 × 10^12^ 1.1 × 10^12^ 8.0 × 10^11^
Sample C (AlN 220 nm)	-	1.7	0.31	2.89	4.0 × 10^9^	7.0 × 10^11^

## Data Availability

All data included in this study are available upon request by contact with the corresponding author.

## Supplementary Materials

The following supporting information can be downloaded at: https://www.mdpi.com/1996-1944/15/23/8602/s1, Figure S1: ꙍ-scan of AlN (sample A) before and after FFA: (a) for the symmetric plane (0 0 0 2), (b) for the skew symmetric plane (1 0 −1 1); Figure S2: ꙍ-scan of AlN (sample B) before and after FFA: (a) for the symmetric plane (0 0 0 2), (b) for the skew symmetric plane (1 0 −1 1); Figure S3. ꙍ-scan of AlN (sample C) after regrowth: (a) for the symmetric plane (0 0 0 2), (b) for the skew symmetric plane (1 0 −1 1).
