# Supplementary material for: Crystalline Quality and Surface Morphology Improvement of Face-to-Face Annealed MBE-Grown AlN on h-BN
Source: Materials (Basel). 2022 Dec 2;15(23):8602. doi: 10.3390/ma15238602 (PMC9736891; doi:10.3390/ma15238602)
Supplement: Supplementary file 1 [file materials-15-08602-s001.zip › materials-2056814-supplementary.pdf]

materials

MDPI

Supplementary Material

Crystalline Quality and Surface Morphology Improvement of Face-to-Face Annealed MBE-Grown AlN on h-BN

Aly Zaiter [1,*], Adrien Michon [1], Maud Nemoz [1], Aimeric Courville [1], Philippe Vennéguès [1], Vishnu Ottapilakkal [2], Phuong Vuong [2], Suresh Sundaram [2,3,4], Abdallah Ougazzaden [2,3] and Julien Brault [1,*]

[1] Université Côte d'Azur, CNRS-CRHEA, French National Center for Scientific Research, 06560 Valbonne, France; adrien.michon@crhea.cnrs.fr (A.M.); maud.nemoz@crhea.cnrs.fr (M.N.); aimeric.courville@crhea.cnrs.fr (A.C.); philippe.vennegues@crhea.cnrs.fr (P.V.)
[2] French National Center for Scientific Research, IRL 2958 Georgia Tech, 2 rue Marconi, 57070 Metz, France; vishnu.ottapilakkal@georgiatech-metz.fr (V.O.); pvuong@georgiatech-metz.fr (P.V.); suresh.sundaram@georgiatech-metz.fr (S.S.); abdallah.ougazzaden@georgiatech-metz.fr (A.O.)
[3] Georgia Institute of Technology, School of Electrical and Computer Engineering, Atlanta, GA 30332-0250, USA
[4] Georgia Tech-Lorraine, 2 rue Marconi, 57070 Metz, France
* Correspondence: aly.zaiter@crhea.cnrs.fr (A.Z.); julien.brault@crhea.cnrs.fr (J.B.)

a)

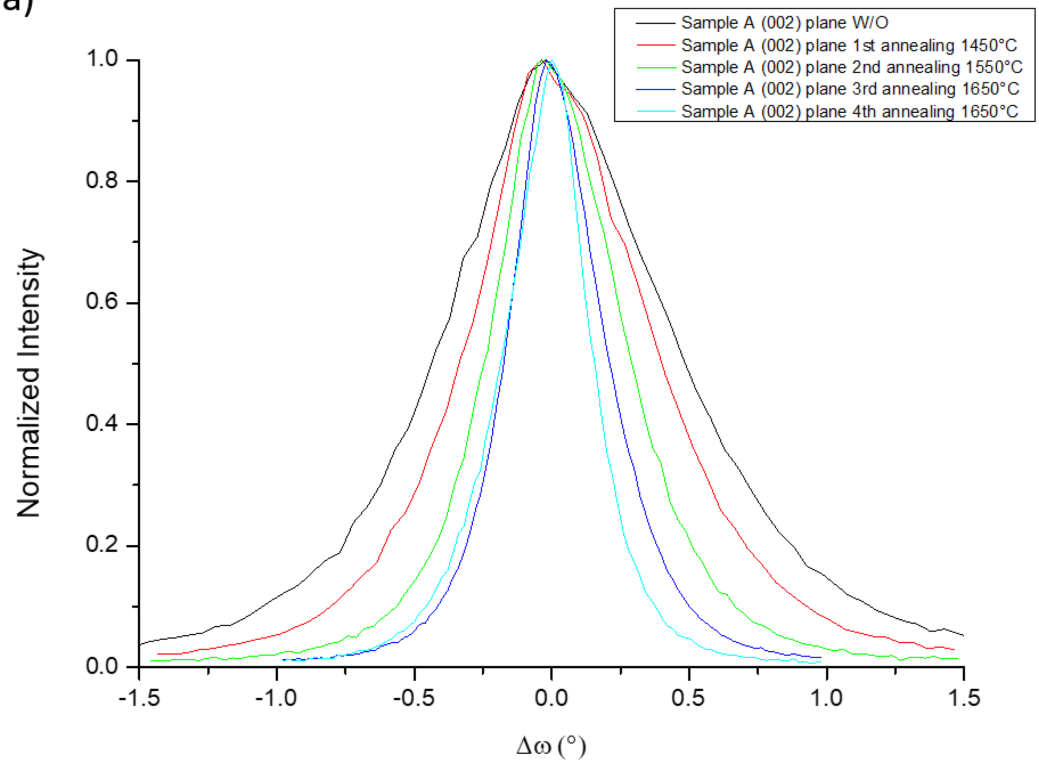

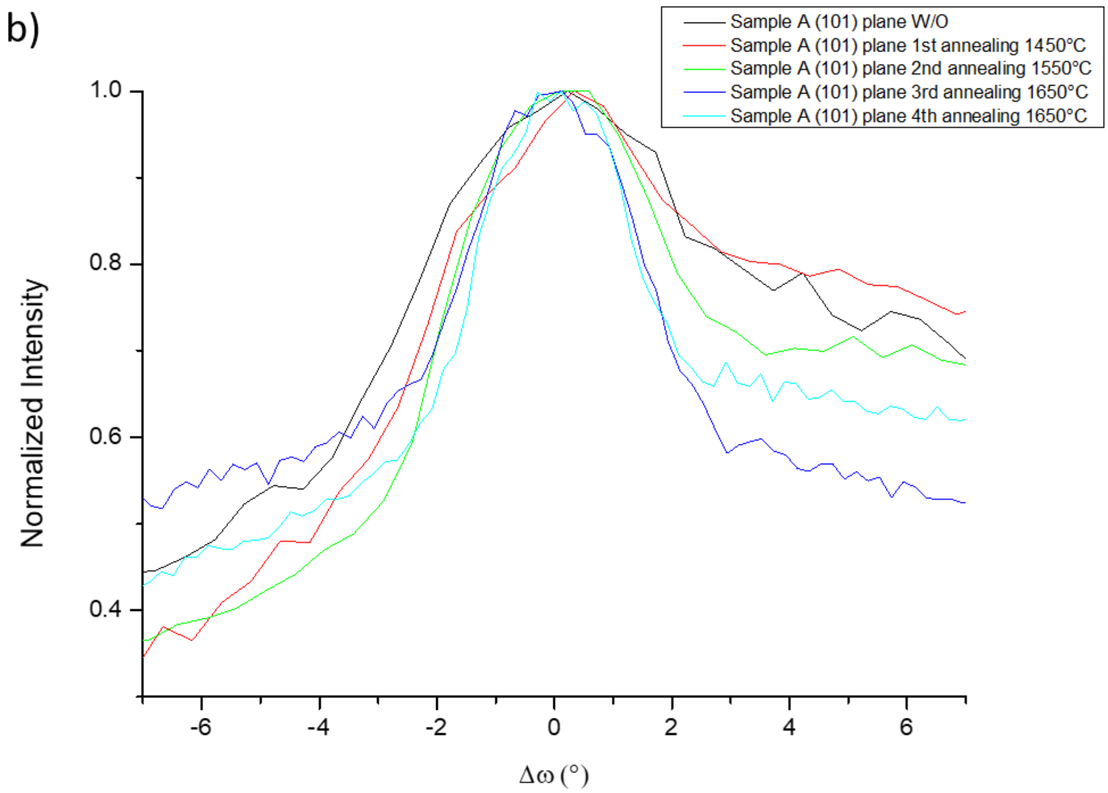

Figure S1. ω-scan of AlN (sample A) before and after FFA: (a) for the symmetric plane (0 0 0 2), (b) for the skew symmetric plane (1 0 -1 1).

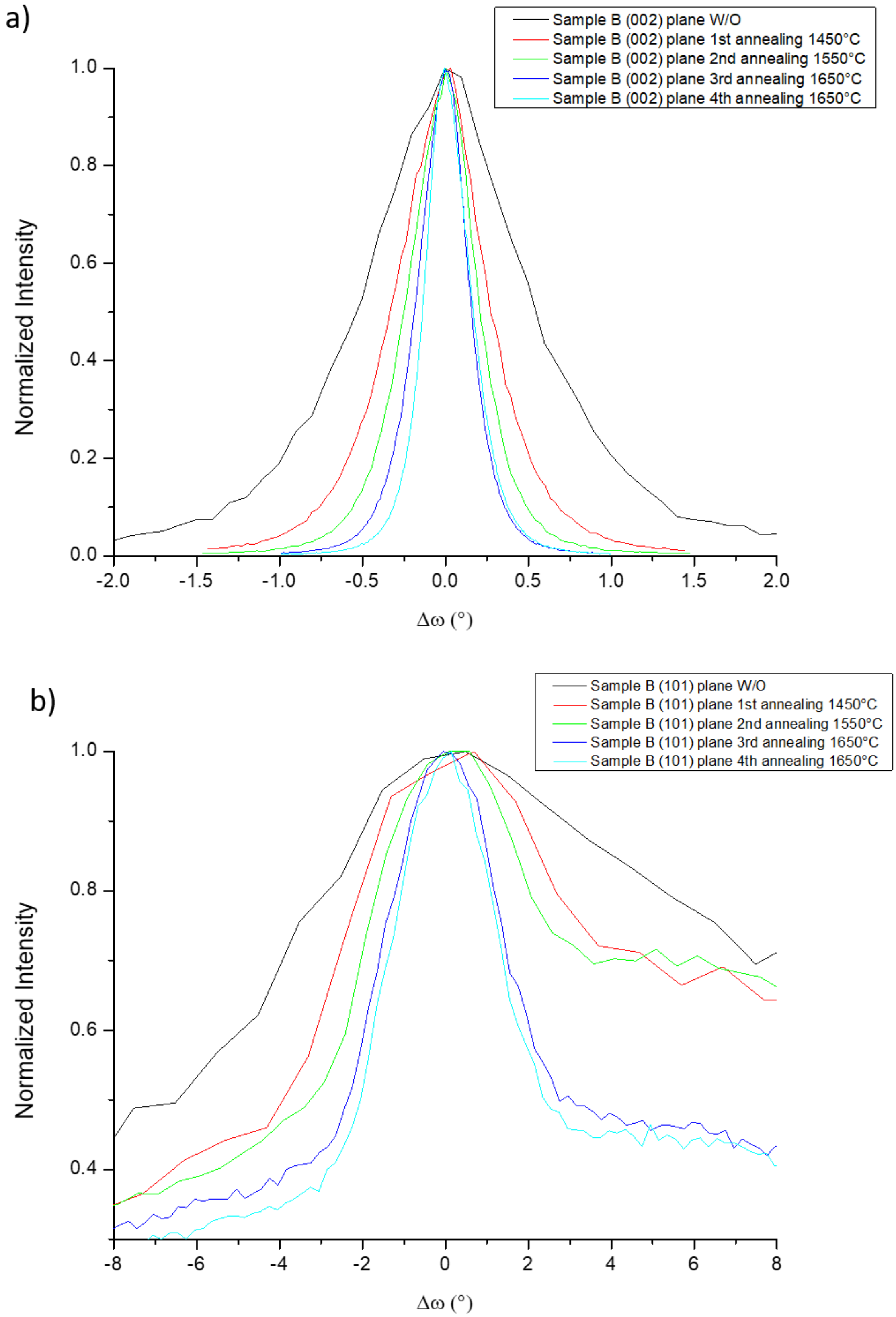

Figure S2. ω-scan of AlN (sample B) before and after FFA: (a) for the symmetric plane (0 0 0 2), (b) for the skew symmetric plane (1 0 -1 1).

a)

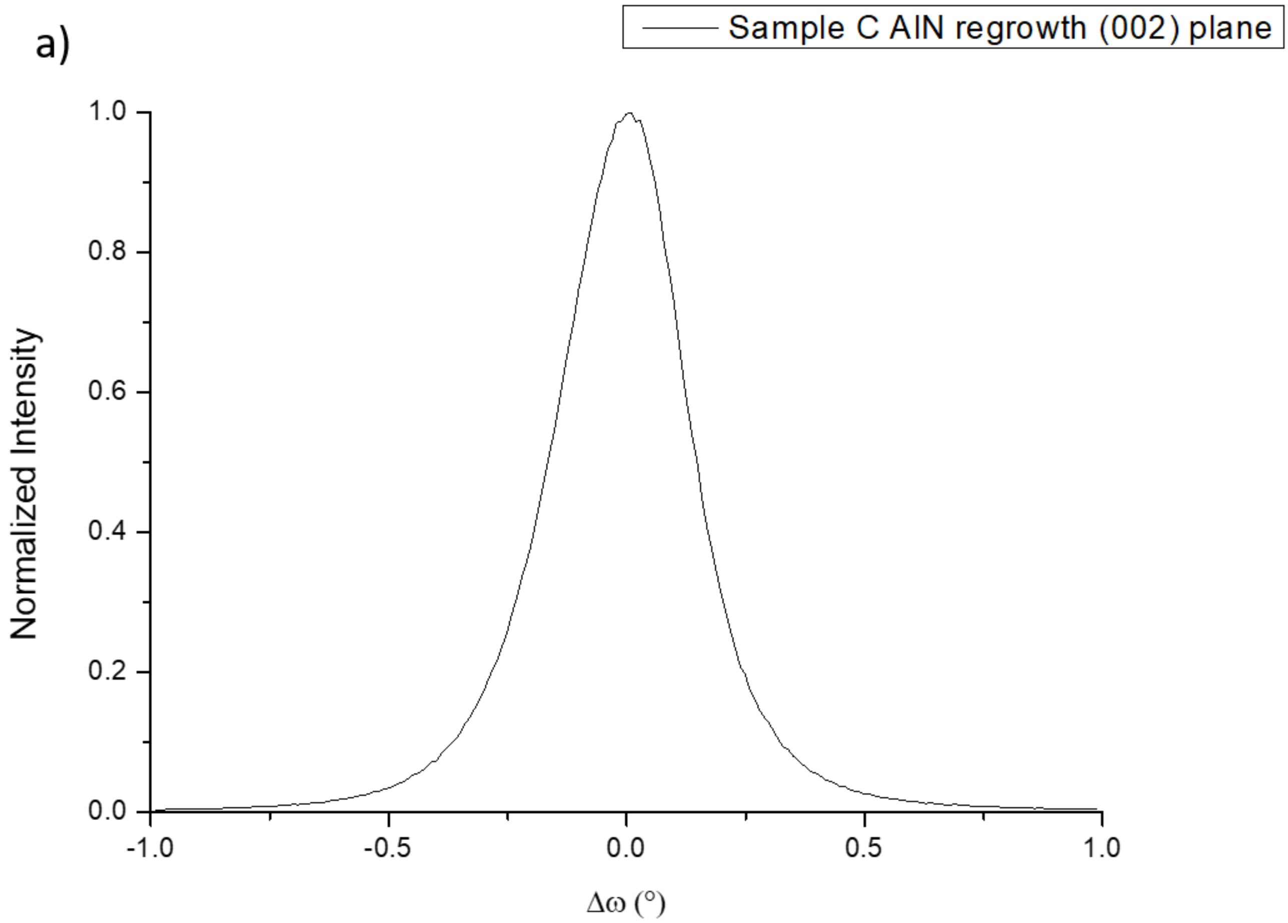
Sample C AlN regrowth (002) plane
Normalized Intensity
1.0
0.8
0.6
0.4
0.2
0.0
-1.0
-0.5
0.0
0.5
1.0
Δω (°)

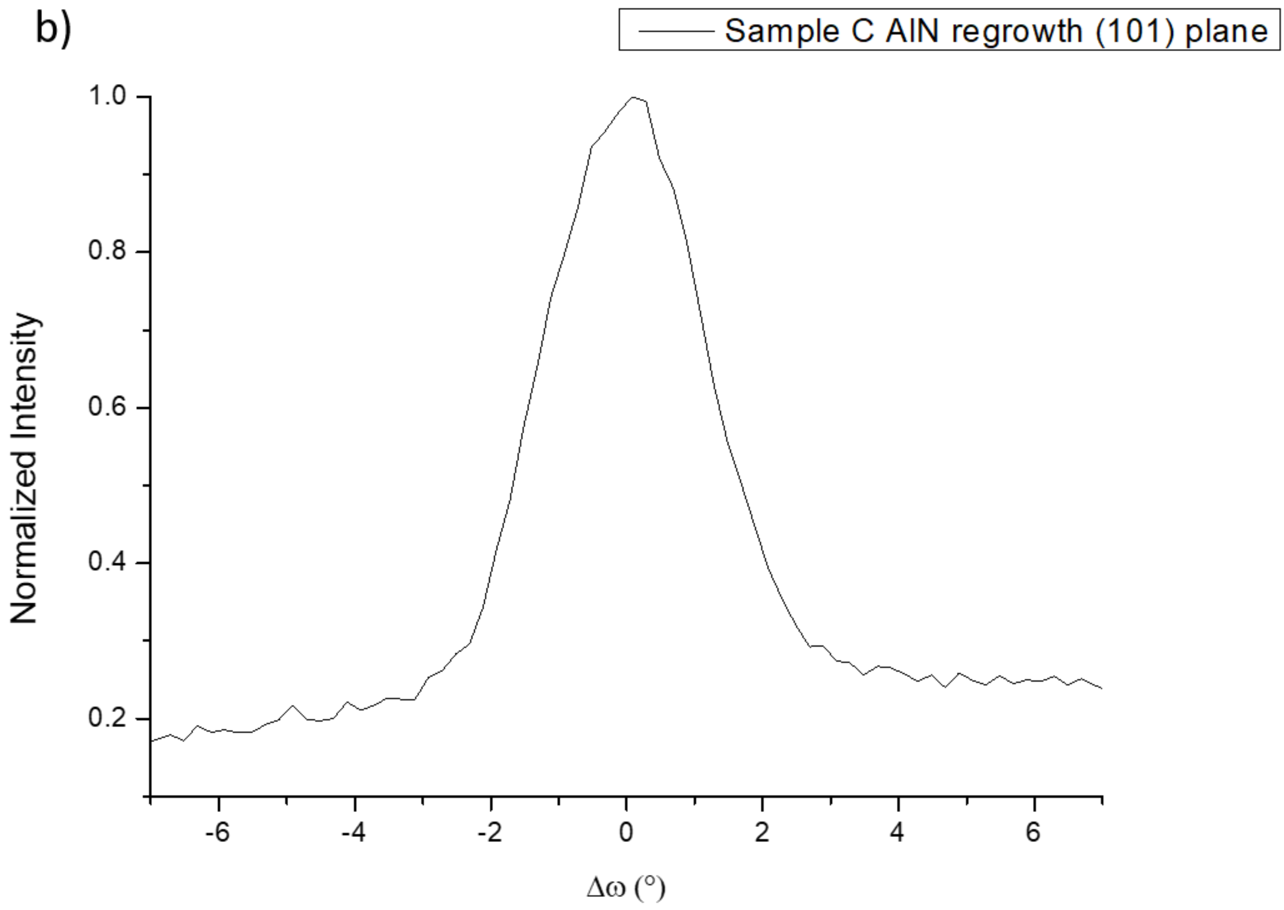

Figure S3. ω-scan of AlN (sample C) after regrowth: (a) for the symmetric plane (0 0 0 2), (b) for the skew symmetric plane (1 0 -1 1).
